# Modelling of fire count data: fire disaster risk in Ghana

**DOI:** 10.1186/s40064-015-1585-3

**Published:** 2015-12-22

**Authors:** Caleb Boadi, Simon K. Harvey, Agyapomaa Gyeke-dako

**Affiliations:** Department of Finance, University of Ghana Business School, Accra, Ghana

**Keywords:** Stochastic, Ecological data, Distribution fitting, Statistical distribution, Negative binomial, Poisson, Fatalities, Fire frequency

## Abstract

Stochastic dynamics involved in ecological count data require distribution fitting procedures to model and make informed judgments. The study provides empirical research, focused on the provision of an early warning system and a spatial graph that can detect societal fire risks. It offers an opportunity for communities, organizations, risk managers, actuaries and governments to be aware of, and understand fire risks, so that they will increase the direct tackling of the threats posed by fire. Statistical distribution fitting method that best helps identify the stochastic dynamics of fire count data is used. The aim is to provide a fire-prediction model and fire spatial graph for observed fire count data. An empirical probability distribution model is fitted to the fire count data and compared to the theoretical probability distribution of the stochastic process of fire count data. The distribution fitted to the fire frequency count data helps identify the class of models that are exhibited by the fire and provides time leading decisions. The research suggests that fire frequency and loss (fire fatalities) count data in Ghana are best modelled with a Negative Binomial Distribution. The spatial map of observed fire frequency and fatality measured over 5 years (2007–2011) offers in this study a first regional assessment of fire frequency and fire fatality in Ghana.

## Background

As population growth rates increase, large numbers of people and infrastructure within urban centres and disaster-prone areas are affected when events such as natural disasters occur (Grid-Arendal and UNEP 2002). Guha-Sapir, Hoyois and Below’s (2013) annual report indicates that in 2012, 357 nature-triggered disasters were registered globally. This was less than the average annual disaster frequency observed from 2002 to 2011 (394), and represented a decrease in associated human impacts of disasters in 2012, compared to previous years. However, between 2002 and 2012, natural disasters killed a significant number of the world’s population. For example, a total of 9,655 people were killed, and 124.5 million people became victims worldwide (Guha-Sapir et al. 2013). Over the past decades, sub-Saharan Africa has experienced more than one thousand disasters, putting at risk many recent development gains due to the vulnerability of its population and economy and their often low capacities to cope with natural hazards (Subramanian and Jha 2010).

Globally, issues of fire-disaster risk have received a lot of attention (Mckenzie et al. 2000; Wang et al. 2005; Cheng and Wang 2008; Bistinas et al. 2014). Most of these studies focused on providing models that helped statistically quantify the likelihood of the frequency of fire for fire prevention (Zhang et al. 2011; Bistinas et al. 2014), whereas others facilitated the understanding of the information needed for fire management decisions (Wang et al. 2005; Taylor et al. 2013). These studies have focused on fitting models for fire frequency, fire interval and burnt areas. Discrete distributions, such as lognormal, Poisson and Negative Binomial Distribution, are the most utilized models for data on fire. They ensure that various models developed for fire data are able to provide timely decisions to mitigate the risk of fire in the various regions of study. These models, which are mostly stochastic, are developed for fire, and are very important for empirical use.

In recent fire outbreaks in Ghana, an analysis of disaster impacts in informal settlements shows that fire causes the greatest loss of life and property in Ghana (Sarpong 2013). Responding to fires, especially in informal settlements, continues to be a daunting task for the vulnerable population because of low capacities developed to prevent fire risks. A summary of natural disaster occurrences indicates that 10 natural disasters occurred from the year 1900 to 2014 (EM-DAT 2014). According to the United Nations Development Programme-UNDP (2013) report, in Ghana, disasters such as fire, pest and flood epidemics occur most often. However, there are limited capacities within government authorities and among communities to cope effectively with these disaster risks. Disaster risk has, therefore, become a commercial issue. Statistically, the forestry sector reveals that the annual loss of revenue from merchantable timber to wildfire is about US$24 million and the cumulative effect of wildfires, an annual loss of 3 % of Ghana’s Gross Domestic Product (GDP) (SDAP 2010). The country loses a lot of money and resources due to numerous fire outbreaks, which include losses from domestic, industrial, institutional, vehicular, commercial, electrical fires and bushfires.

It can be inferred that the relationship between fire occurrence and its socioeconomic impact is of great significance because of which Wang et al. (2005) argue that it requires prediction, fire protection and fire risk assessment. Rossel (2012) refers to such situations as forecastable. The UNDP (2013) recommends that minimising uncertainties of disaster risk requires that necessary knowledge prior to the occurrence of the disaster through monitoring, analysing and forecasting of the possible events and their impact is available. This will ensure that imminent situations will be communicated and disseminated effectively to local capabilities. By these actions, the people at risk would be able to respond to the warnings received.

Global and regional studies have focused on fitting models for fire frequency, fire interval and burnt areas (Bistinas et al. 2014; Júnior et al. 2014; Jiang et al. 2012). Other studies have also gone a step further to provide models that will help to statistically quantify the frequency of the likelihood of fire for fire prevention (Mckenzie et al. 2000; Cheng and Wang 2008; Syphard et al. 2008; Zhang et al. 2011; Bistinas et al. 2014), while others have provided understanding for the information needed for making fire management decisions (Wang et al. 2005; Taylor et al. 2013). On the provision of better-fit models to fire count data and risk assessment and predictions, Mehrannia and Pakgohar (2014) argue that the distribution fitting procedure is best used to select a statistical distribution. This work, in order to avoid choosing any incorrect model that will compromise the ability of the system to generate and project time-leading decisions, adopted the distribution fitting procedure. It seeks to contribute to existing literature by identifying a base distribution for quantifying the likelihood and magnitude of losses arising from fire on the various administrative regions in Ghana. It empirically fits an appropriate statistical distribution to fire count data, assesses the likelihood of a fire risk affecting properties and the economy, and also determines the fatality that can be expected through fire occurrence.

Early Warning System (EWS) is a set of capacities needed to generate and disseminate timely and meaningful warning information to enable individuals, communities and organisations threatened by a hazard to prepare and to act appropriately and in sufficient time to reduce the possibility of harm or loss (UNISDR 2009; IFRC 2013). Several studies on the occurrence of hazards or disasters around the world focus on providing early warning systems as an integral part in risk communication (Marvin et al. 2013; Souaré and Handy 2013; Mayhorn and McLaughlin 2012). EWS, globally, has received acclaim, and is used to provide a preparatory ground toward any unforeseen occurrences. From the review of studies (e.g. Basher 2006; Davis and Karim 2008; Huang and Chou 2008; Salzano et al. 2009; Jin and Lin 2011; Lautze, et al. 2012; He et al. 2012; Koyuncugil and Ozgulbas 2012; Mayhorn and McLaughlin 2012; Dokas et al. 2013; Marvin et al. 2013; Souaré and Handy 2013), it is observed that most early warning systems focus on providing an early warning sign identification of natural hazards, financial risk, data mining risk detection and societal risk detection, using ecological data, such as the number of conflicts, the number of voters, the number of fires, etc.

Methods used to predict early fire signs in the literature are dissimilar due to how data is collected on fire from various macro-economic environments, which happen to be different in form. It is asserted that fire data in previous studies is complex (Moritz et al. 2009), and include weather and fire history, fire ignition and fire spread, involving censored and counted data. Due to the complex nature of data on fire occurrence in various areas, several methods and models have arisen to help manage fire risk. In a critical assessment, the deterministic and stochastic methods have been discovered to be the two major models frequently used by ecologists in modelling fire. The deterministic method incorporates physical mechanisms for fire spread and fire growth but does not allow for a stochastic variability of the output either by varying the initial conditions or varying the weather or fuel type to see how fire will grow or spread (Boychuk et al. 2009). Bolker (2008) argues that a purely deterministic model allows for only qualitative comparisons with real systems. However, the stochastic model incorporates noise or randomness that is not captured by the deterministic models. It is therefore necessary to specify a reasonable probability distribution for the variations often identified in count data. The study uses the stochastic processes to help achieve the study objectives.

The stochastic process, compared with the deterministic approach, is more useful in handling count data. It helps identify the variability that exists in count data due to environmental variation and provides an appropriate model for determining the likelihood of future uncertainties (Bolker 2008). A random accumulation process nature and binary indicator of the frequency of fire events could be identified using catastrophic models. This phenomenon of occurrence of the event varies to some degree, becoming unpredictable as time goes on and, therefore, requires the use of probability distributions to scientifically model the uncertainties involved in order to make informed judgments. The Poisson Distribution is a popular distribution for analysis of count data (Gardner et al. 1995). Mandallaz and Ye’s (1997) study presents a general statistical methodology for the prediction of forest fires in the context of Poisson models. In most of the ecological data, which are mostly count data, stochastic models such as the negative binomial, seen to encapsulate the over-dispersion parameter that is not found in the Poisson Distribution, is widely identified. Bolker (2008) suggests that due to its unfamiliarity, ecologists tend to use deterministic building blocks (e.g. linear or Michaelis–Menten functions) rather than stochastic building blocks (e.g. the negative binomial or gamma distributions).

## Methodology and data

This section presents the distribution fitting procedure that is used to select a base statistical distribution for fire count dataset identified in Ghana. The distribution fitting method is preferred because of the desire in this work to find the best distribution type, especially in cases where there is little or no information about the base distribution pattern in the data (Mehrannia and Pakgohar 2014). However, to our knowledge, no base distribution for predicting fire occurrences has been used in Ghana. Using the steps in modelling count data, this study fits statistical distributions with the past events of fire disaster data. In order to appropriately fit the base distribution to the fire count data, the study judiciously considered a number of distributions that possibly fit the data and estimated the model parameters, based on the goodness of fit test (Log-likelihood statistics, AIC, BIC) and subsequently selected the best fit model for the data set.

### Selection of certain family of distributions and estimating model parameters

The stochastic method provides the most comprehensive depiction of the likelihood of losses from extreme events. It is, therefore, enlightening to examine some models that are used for count data.

The most utilized statistical distribution for analysis of count data is the Poisson Distribution (Bolker 2008; O’Hara and Kotze 2010). The Poisson model assumes an underlying constant rate of event and provides the count of events over a fixed amount of time at risk. The Poisson model is appropriate for observing fire count data. The Probability Mass Function (PMF) of observing any specific count *y*, over time *t* for a given area with average frequency μ per unit time is given as;

$$\Pr ( {Y = y;\mu t} ) = \frac{{(\mu t)^{y} e^{-\mu t}}}{y!}\;\;\;{\text{where}} \quad y = 0,1,2, \ldots ,\mu >0$$

and *μt* is called the rate of the Poisson process. When data does not fit the Poisson Distribution, it is typically as a result of over-dispersion. It is appropriate to deal with count data of over-dispersion using a negative binomial model (Hilbe 2014).

As a result of the over-dispersed nature of count data (Linden and Mantyniemi 2011), the study additionally considered the negative binomial as a benchmark technique in modelling the stochastic variations of fire count data. The negative binomial is selected because it allows for various quadratic mean–variance relationships that are involved in the count data. Negative Binomial Distribution accounts for the practices, which lead the Poisson model to over-dispersion and under-dispersion and, therefore, provides another parameter that quantifies extra Poisson variation (Lawless 1987). The negative binomial model offers a far wider range of variability than the Poisson. If a non-negative discrete random variable X has a Negative Binomial Distribution with parameters *k* and $\mu$. denoted by NB (*k*,$\mu$), then the Probability Mass Function (PMF) of the variable X is expressed as;

$$f\left( {X = x} \right) = \frac{\varGamma (x + k)}{\varGamma (x + 1)\varGamma (k)} \left( {\frac{k}{k + \mu }} \right)^{k} \left( {\frac{\mu }{k + \mu }} \right)^{x} \,\,\,{\rm for}\,x = 0,1 \ldots$$

The negative binomial regression model with no covariates has mean *μ*, in the Poisson model, but the variance is *μ* + *μk*^*2*^, thus allowing the variance to exceed μ. The parameter k is called size (over-dispersion) parameter because it is mathematically equivalent to *n* (number of failures) in the failure-process parameterization of the Poisson Distribution.

Although the above distributions have proven appropriate to well model count data, the distribution fitting procedure is employed to select a statistical distribution for the data so as to avoid choosing any incorrect model. The distribution fitting procedure provides the study with a base distribution for identifying fire, their parameters indicating the possible occurrence and a confidence interval providing the lowest and highest probability of producing the observed data.

In estimating the model parameters, the method of Maximum Likelihood Estimation (MLE) which attempts to find the values of the true parameters that are most likely produced by the data observed is used. It is the most versatile method for fitting parametric statistical models to data (Cousineau et al. 2004).

Fire count data (monthly fire occurrence and corresponding fatalities) from the Ghana Open Data Initiative from the administrative regions in Ghana are used in this study to help develop EWS on fire. The fire count data from Ghana from 2007 to 2011 consist of the monthly number of rescue injuries, death and fire frequency in all the regions in Ghana.

## Empirical analysis and discussion

To illustrate and identify the best fit distribution, we used an exploratory analysis of data and diagnostic test probability plots to demonstrate certain families of distribution to the fire count data.

Exploratory fire count data in various regions which are monthly reported are previewed in this section. To provide exploratory analysis of the fire occurrence data we present histogram plots and fire sequence analysis of count data. The histogram plots with an overlaid vertical bar presents summaries of data among regions.

**Fig. 1:**
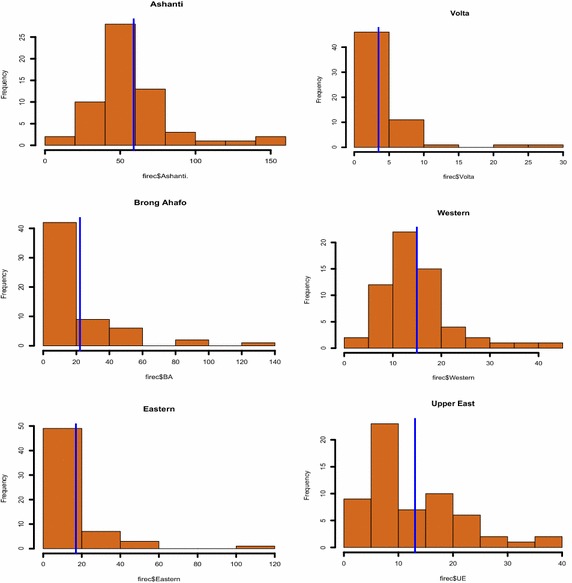
Fire occurrence histogram plots for regions with a vertical bar overlaid showing the mean occurrence

**Fig. 2:**
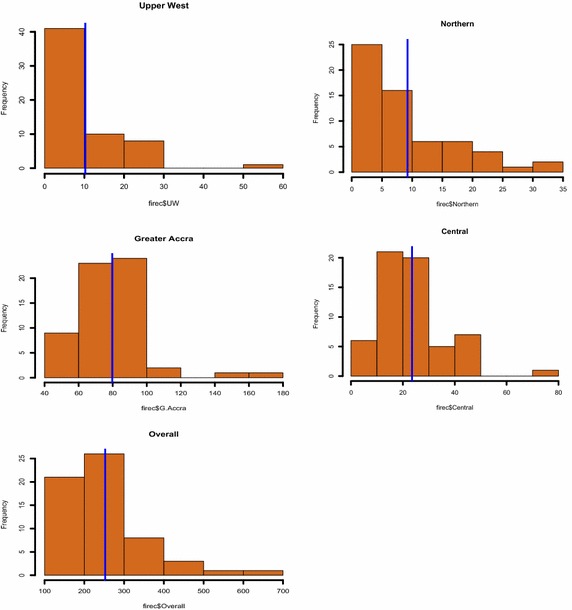
Fire occurrence histogram plots for regions with a vertical bar overlaid showing the mean occurrence

**Fig. 3:**
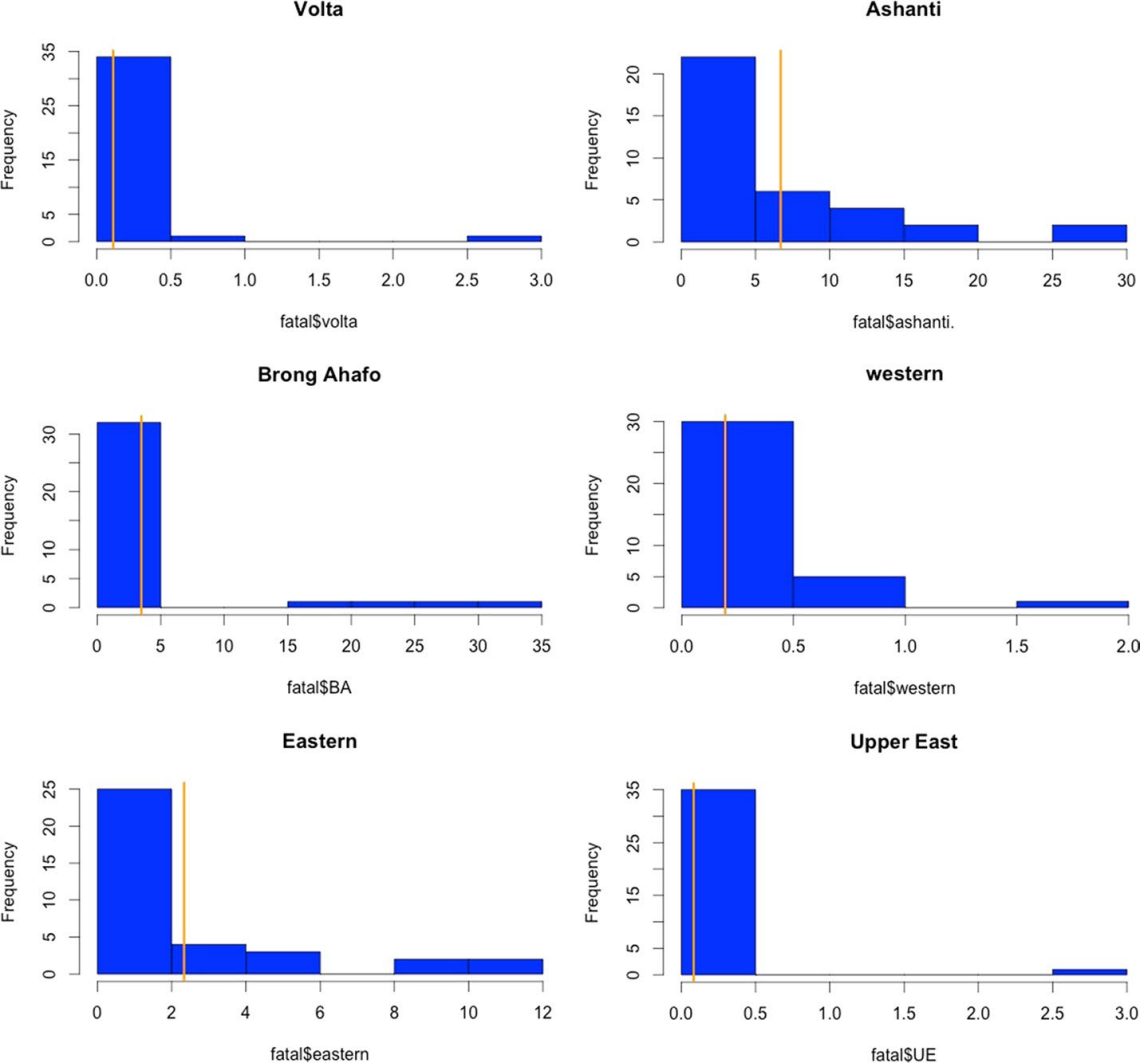
Fire fatality histogram plots for regions with a vertical bar overlaid showing the mean occurrence of death through fire

**Fig. 4:**
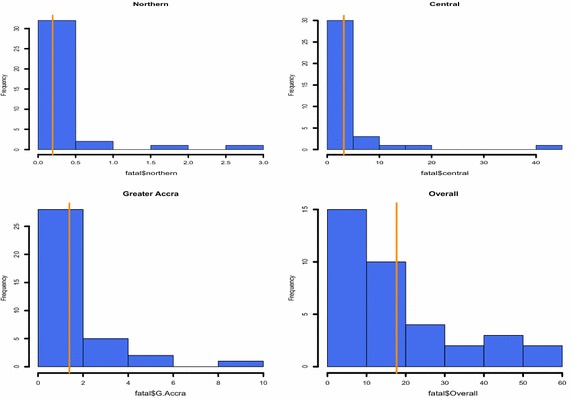
Fire fatality histogram plots for regions with a vertical bar overlaid showing the mean occurrence of death through fire

As shown by the histogram plots for fire occurrence (Figs. 1, 2), the Greater Accra Region records the highest monthly fire occurrence. It is not surprising to see that Accra experiences the highest monthly fire occurrence frequency since it is the most populous city in Ghana. Also, summaries of the economic impact of fire fatalities are shown by the histogram plots (Figs. 3, 4). It is observed that the highest monthly fire fatality is recorded in the Ashanti Region, followed by the Brong Ahafo Region. Shown by the histogram for all other regions provides a visualization of fire occurrence and fire fatality and thereby giving a fair idea about the data.

**Fig. 5:**
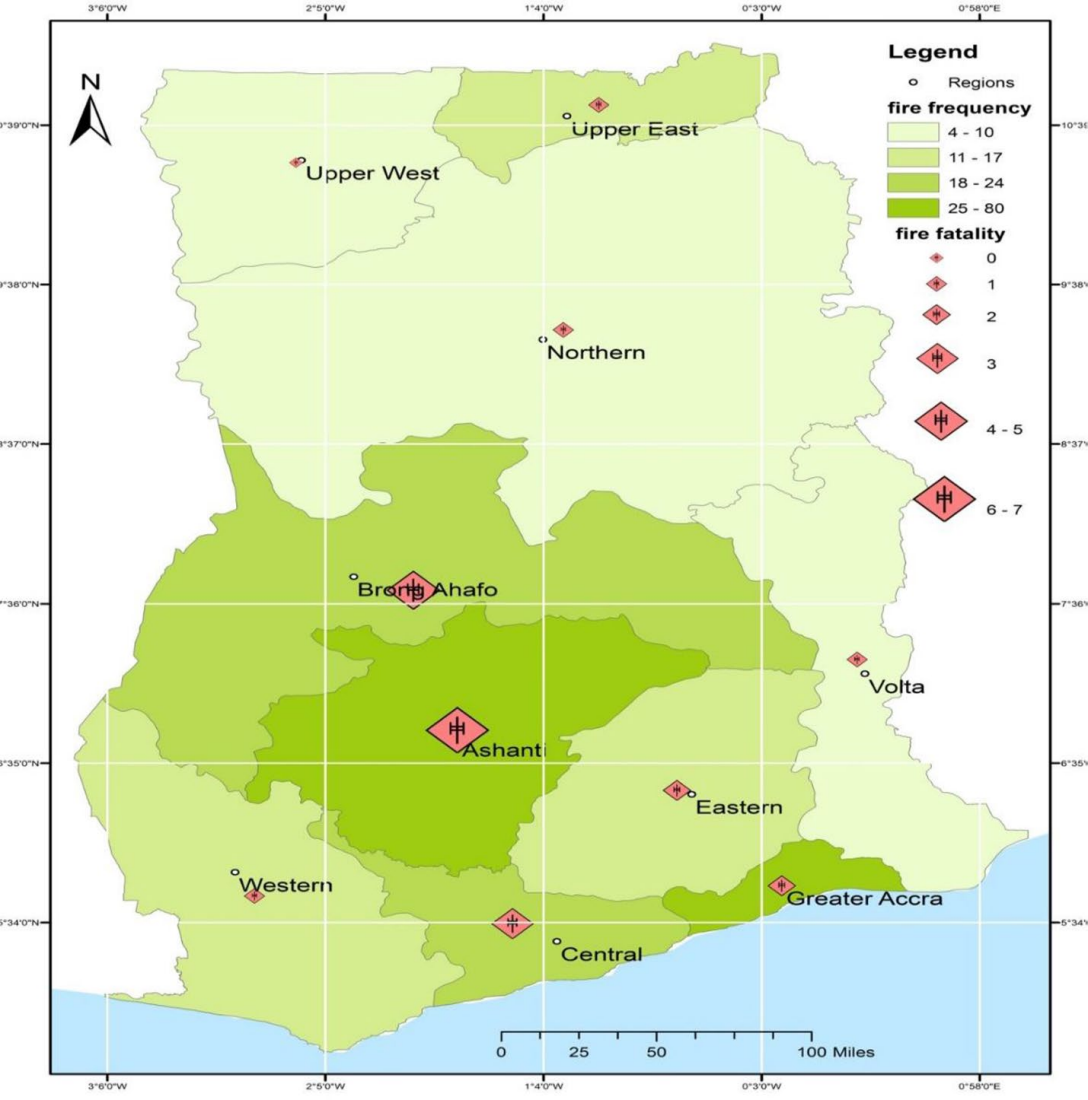
Spatial map of observed fire frequency and fatality for regions in Ghana. The fire frequency indicates the observed monthly frequency of fire throughout the country. The fire fatality also indicates the deaths that result from fire occurrences among the regions

The plots above are fire frequency and fatality sequence plots indicating how fire and death through fire occur in months in Ghana. It may be observed that the highest frequency of fire occurs during the first months of the year and reduces gradually to the month of August. However, as shown from the graph during the period of September, the frequency of fire rises till the end of the year. Considering the fatalities that arise as a result of the fire it is indicative that the monthly fire fatalities increases or decreases in the country. It is observable that the highest fire fatalities were recorded during the month May. January, May and the month November are also seen to have higher fatality resulting from fire while fatalities for the rest of the months are low. The visual representation of observed fire fatality and fire frequency in the spatial map below indicates how fire occurrence and fatality through fire are distributed along the regions of Ghana. Fire occurrences observed over the period of the study are spatially plotted providing an indication of the severity of fire frequency and fire fatality on the economy (Fig. 5).

**Fig. 6:**
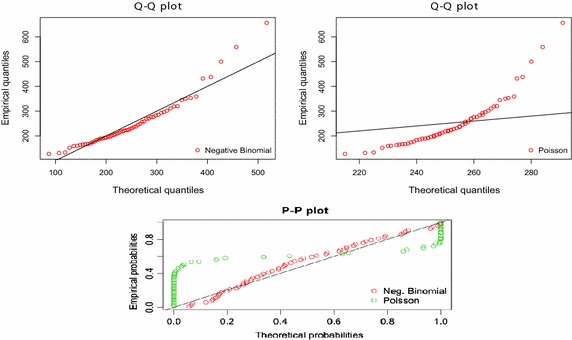
Q–Q and P–P plot for fire data distribution

In selecting the family of distribution for the dataset, an empirical density cumulative distribution graph and the quantile–quantile (Q–Q) of theoretical distributions from R-software is used. The observatory Q–Q and probability–probability (P–P) plot (Fig. 6) identifies the Negative Binomial Distribution and the Poisson Distribution as the candidate models after testing a wide range of distributions of fire data. The Q–Q plots provides visualized tool for inspecting the fit of the candidate models, comparing the fitted and empirical distributions in terms of the dimensional values of the dataset. The closely plotted points are to the line of best fit, the better the model. The P–P plot is also indicative; it fits the three common distributions to the fire count data, indicating which of the statistical distributions best model fire count data.

Given the three candidate models of fire count data, making estimates of the model parameters using maximum likelihood estimation is achieved based on the empirical data. Achieng (2010) suggests that obtaining the parameters of the distribution literally means the statistical distribution has been fitted to the dataset. Table 1 shows the parameter estimates from the two models of fire frequency data. The Negative Binomial (*k* and *μ* parameters) employs more general specifications and a standard choice for a basic count data model. The parameters obtained for the statistical distribution for each region is previewed with their probabilities in Table 1. The 95 % confidence interval contains the true parameter with a probability of 0.95.

**Table 1:** Parameter Estimates with confidence interval for fire frequency

Regions	Negative binomial	
	k	μ
Ashanti	4.03 ± 6.83	59.00 ± 6.83
Volta	0.5 ± 0.24	3.48 ± 1.31
Brong Ahafo	0.65 ± 0.25	22.18 ± 7.08
Western	4.70 ± 2.31	14.97 ± 2.00
Eastern	2.29 ± 0.92	16.92 ± 3.02
Upper East	2.56 ± 1.14	13.05 ± 2.26
Upper West	1.74 ± 0.71	10.23 ± 2.12
North	1.35 ± 0.58	9.23 ± 2.16
Central	2.85 ± 1.21	23.51 ± 3.73
Greater Accra	19.53 ± 8.60	79.75 ± 5.10
Overall	8.09 ± 2.92	252.34 ± 22.81

*Overall* Fire data on the whole economy

Pertaining to the fitted statistical distributions, it is observed that for the Negative Binomial Distribution, Greater Accra received the highest mean of monthly occurrences of fire with the highest over-dispersed parameter of 19.53. This indicates that the Region is susceptible to a higher occurrence of fire monthly. Hence, the occurrence of fire is riskier in Greater Accra than in other regions. Although some regions have higher expected fire occurrences, the over-dispersed parameter indicates how risky the occurrences are within months. We can infer that monthly variations of fire occurrence for the Western Region show that it is the second-highest risk-prone region with higher changes in the monthly fire occurrence. However, considering variations of fire occurrence, it is observed that the Ashanti Region is also a highly risky region.

**Fig. 7:**
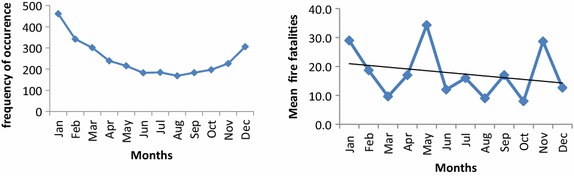
Observed fire frequency and fire fatality sequence plot

Similarly, concerning the impact of fire fatalities (deaths through fire occurrence) on the economy, fire fatalities distribution profiled in Table 2 captures the parameter estimates from the two models considered. These models were selected based on theoretical and empirical judgments using several probability graphs, shown in Fig. 7. The Negative Binomial (r and θ parameters) and the Poisson Distribution (μ) are fitted to the fire fatality data on the regions and overall. The parameters obtained from fire fatality from the various statistical distributions are shown in the Table 2 below.

**Table 2:** Parameter Estimates with confidence interval for fire fatality

Regions	Poisson	Neg. binomial	
	μ	k	μ
Ashanti		1.03 ± 0.57	6.29 ± 2.31
Volta		0.05 ± 0.11	0.11 ± 0.20
Brong Ahafo		0.21 ± 0.13	3.47 ± 2.55
Western	0.19 ± 0.14		
Eastern		0.41 ± 0.28	2.33 ± 1.29
Upper East		0.02 ± 0.04	0.08 ± 0.24
Upper West	ND	ND	ND
North		0.13 ± 0.23	0.19 ± 0.23
Central		0.27 ± 0.16	3.19 ± 2.10
Greater Accra		0.47 ± 0.39	1.39 ± 0.76
Overall		1.48 ± 0.70	17.67 ± 4.94

*Overall* fire data on the whole economy, *ND* no data

The parameter estimates fitted by the statistical distribution for fire fatality indicates that the Ashanti Region received the highest mean of monthly occurrences of death through fire with an over-dispersed parameter of 1.032. Considering the over-dispersion parameter of the negative binomial, the Western Region is the riskiest region for which death through fire varies widely within months. Although some regions, such as Central, Brong Ahafo and Eastern regions recorded high fatality through fire, the over-dispersed parameter indicates less riskiness in the monthly death rate through fire compared to the Western Region. Again, in terms of the variations of fire fatality amongst regions, Brong Ahafo is the riskiest region, followed by the Central and Ashanti regions. Variations amongst the fitted distribution are less communicated. Therefore, we used various goodness of fit tests to select the best model for fire fatality in order to arrive at better decisions.

In the selection and testing of goodness of fit of the estimated parameters, the Log-likelihood statistics, the Bayesian Information Criterion (BIC) and Akaike Information Criterion (AIC) are used to choose the best model out of the candidate models. A specified likelihood of a parameter points toward a possible result. Log-likelihood forms the basis for which we selected the best fit model. It is worthwhile to take decisions that may lead to possible results if only the Log-likelihood statistics is high enough. Meanwhile, the Bayesian Information Criterion (BIC) and Akaike Information Criterion (AIC) minimize prediction error and freely estimated parameter. This work seeks to identify a model that does not over fit the data with too many parameters while simultaneously accurately modelling the data. In a model selection application, the optimal fitted model is identified by the minimum value of the BIC and AIC. Based on these tests and the above Q–Q and P–P plots, various regional models for fire occurrences and fatality are fitted.

Shown by the values of the log-likelihood statistics from the R-software (Table 3), the Negative Binomial Distribution is fitted for fire occurrence frequency on the regions. The high values, compared with other models, indicate the likelihood of the parameter of the various fire frequency data. Hence, the over-dispersed Negative Binomial Distribution is the best fit of the three candidate distributions for the regions. The high values of log-likelihood statistics and minimum AIC and BIC values chooses the Negative Binomial distribution. This shows that the unknown true model fits the Negative Binomial Distribution for all the fire frequency data. We can, therefore, argue that the fire count data for all regions in Ghana, which follow a Negative Binomial Distribution, the closest to the true model. This means that fire count data on the various regions are better modelled by the failure-process parameterization of the negative binomial; a good phenomenological description of a patchy or clustered distribution with no intrinsic upper limit that has more variance than the Poisson (Bolker 2008).

**Table 3:** Log-likelihood, AIC and BIC Statistics on fire frequency parameter

Regions	Neg. Binomial		
	Log-likelihood	AIC	BIC
Ashanti	−287.45	578.89	583.08
Volta	−139.02	282.04	286.23
Brong Ahafo	−244.67	493.33	497.52
Western	−206.02	416.05	420.24
Eastern	−224.06	452.12	456.31
Upper East	−208.25	420.51	424.70
Upper West	−198.95	401.90	406.09
North	−195.61	395.23	399.42
Central	−240.37	484.73	488.92
Greater Accra	−263.98	531.95	536.14
Overall	−352.56	709.12	713.31

Fatality through fire has also been analysed in Table 4 using the log-likelihood, AIC and BIC statistics obtained from the parameter estimates. It is shown that Poisson received the least value of the log-likelihood value for data coming from the Brong Ahafo Region, while data from the Upper East Region showed the highest value of log-statistics fitting Negative Binomial Distribution of the fire fatalities observed. In this regard, the model fitted by the log-likelihood is confirmed. The Negative Binomial is noted to better model fire fatality. However, the Poisson Distribution was fitted for fire fatality data from the Western Region. The selection of this model for fire data is consistent with Xiao et al.’s (2015) study, confirming that fire occurrence count data models are likely to be dispersed and frequently contain an excess of zero counts. It is not surprising, therefore, that the NB model provided a more compelling and credible inferential for the count data. This, indeed, is an indication that the prediction achieved through the Negative Binomial model provides a more compelling and credible inferential basis for fitting actual fire occurrence (Xiao et al. 2015). These results are consistent with the plots shown in Fig. 7, which shows that negative binomial is the best model for count fire data, in comparison with the other candidate models of this study.

**Table 4:** Log-likelihood, AIC and BIC Statistics on Fire Fatality Parameter

Regions	Poisson			Neg. binomial		
	Log-likelihood	AIC	BIC	Log-likelihood	AIC	BIC
Ashanti	−172.54	347.09	348.67	−107.00	218.01	221.18
Volta	−14.58	31.16	32.74	−10.61	25.22	28.38
Brong Ahafo	−213.08	428.16	429.75	−70.36	144.73	147.90
Western	−19.16	40.31	41.90	−19.08	42.16	45.33
Eastern	−108.88	219.76	221.35	−70.05	144.10	147.27
Upper East	−12.25	26.49	28.08	−6.79	17.58	20.74
Upper West	ND	ND	ND	ND	ND	ND
North	−20.95	43.90	45.48	−17.51	39.02	42.19
Central	−192.42	386.84	388.42	−72.27	148.54	151.70
Greater Accra	−74.14	150.29	151.87	−56.99	117.99	121.16
Overall	−295.20	592.39	593.97	−139.13	282.26	285.43

*Overall* fire data on the whole economy, *ND* no data

It is proven by the log-likelihood statistics, AIC and BIC goodness of fit test that the Negative Binomial Distribution that emerged is a better model for observed fire data amongst the three candidate models. The quantile–quantile plots for the fire data show that, theoretically and empirically, the NB and the Poisson are fitted for the fire data. The probability (P–P) plots for overall fire data of the fitted distribution constructed above indicate the goodness of fit of the Negative Binomial Distribution. The P–P plot gives useful comparisons for probability distributions that have a nearby or an equal location. The Probability plots in (Fig. 7) above depict a very good fit for a negative binomial with almost all the data points falling onto or around the reference line. We can, therefore, confidently argue that the Negative Binomial Distribution does provide the correct statistical model for fire frequency data on the various regions. It is also indicative that the over-dispersed nature of count fire data in Ghana is better modelled with the two parameter Negative Binomial Distribution.

Numbers containing the most plausible values for our population parameter are simulated through bootstrapping and sampling parameters fitted by the distribution. Stochastic variates from the probability distributions helps make predictions. Though bootstrapping is a better tool for confidence interval estimation, we have further determined how much confidence to place in the parameter estimates. The 95 % confidence interval provides an interval that contains the actual true parameter value of fire fatality and fire frequency with probability 0.95. The parameters identified above are simulated and the various confidences show the true value where the actual parameter lies. Based on the various confidence intervals provided in Table 1, we will be able to determine subsequent fire occurrences with the fitted model within the lower and upper limit of the parameter estimated across the various regions. The Negative Binomial Distribution and the Poisson are fitted for the fire loss (fire fatality) data from the various regions previewed in Table 2. Equally presented in Table 2 are the various predictions of the parameters, using a 95 % confidence interval.

The empirical results above illustrate the modelling process in the estimation of the distribution of loss through fire and the frequency of the occurrence of fire. After identifying the best fit distributions of the variables the study took a step further by predicting the parameters estimated using confidence intervals. These are the principles by which the study has modelled the fire occurrences and fire fatality in the ten administrative regions of Ghana, using data from the Ghana Open Data Initiative (GODI). The results of this work demonstrate that the severity of fire frequency and fatality on the economy are best modelled with similar distribution while the distribution of loss (fire fatalities) are indeed better modelled with different distributions. We observe that the fire frequency data for most of the regions follow a Negative Binomial Distribution. It is indicated by the various statistical tests (log-likelihood, AIC, and BIC) that the Negative Binomial and the Poisson Distributions better model the various fire count data in the administrative regions of Ghana. We argue that the Negative Binomial model is good for modelling stochastic variability of count data around a theoretical expectation, taking into account the over-dispersion that arises from the data. With regard to the over-dispersed nature of the count fire data, the parameter estimates provide a nearly-identical frequency-based maximum likelihood estimates of the model. Standard errors of these parameters also provide estimates of uncertainty of the parameters that are of interest to the model. Parameters are predicted with the 95 % confidence interval tables provided in the study. The spatial map in this study provides a more readily interpretable visualization of the severity of observed fire frequency and fatality on the economy of Ghana. The spatial map in this study provides patterns that are spatially comparative of fire risk, a first kind of a regional assessment in Ghana.

## Conclusion

From the results of the study, it is posited that fire frequency and loss (fire fatalities) count data in the regions in Ghana are best modelled with similar distribution. Fire risk events in Ghana have been modelled with negative binomial. This is identified in the literature to be the best for modelling stochastic variation of ecological count data around a theoretical expectation data by taking into account the over-dispersion that arises from the data. Although the Poisson Model is the simplest count data model, it is highly restrictive, as the variance of the outcome is equal to its expectation. Fire count data exhibit over-dispersion. Therefore, the Negative Binomial Distribution that offers a dispersion parameter is argued to cogently explain positive count fire data. In this study, our negative binomial model was limited because it did not contain covariates. Future studies should focus on building models that capture several local covariates among regions. Considering the events of fatality and fire occurrences, we observed from the spatial graph and the sequence plot that frequency of fire occurrences and fire fatality are closely related. Future studies should focus on determining the relationship of capacities built among these regions to help mitigate fire occurrences. An extension of this work will focus on studying whether the efforts of Government of Ghana have succeeded in fire prevention.

## Recommendation

Efforts need to be supported by researchers, fire officers and risk managers for further improvement and more effective fire management. Data collected on fire should be more robust in order to encapsulate certain activities that lead to fire outbreak. The National Disaster Risk Management and Ghana Fire Service should deploy resources to various areas that are perceived to have high rates of fatality through fire. Based on the predictions of the statistical models, we suggest that Government and stakeholders should make available necessary equipment to help fight fire in various vulnerable areas in the regions.

## Appendix: Ecological map of Ghana

This appendix provides an overview of the ecological zones of Ghana. The map gives an ecological description of the country providing coverage of the Coastal scrub and grassland, Guinea savannah, moist-semi deciduous forest, rain forest, strand and mangrove, and the Sudan savannah zones in Ghana.
